# Perioperative risk factors for survival outcomes in elective colorectal cancer surgery: a retrospective cohort study

**DOI:** 10.1186/s12876-021-01757-x

**Published:** 2021-04-13

**Authors:** Xing-Xing Liu, Jun Su, Yuan-yuan Long, Miao He, Zhao-Qiong Zhu

**Affiliations:** 1 Soochow University Medical College, SuzhouJiangsu Province, 215000 China; 2 Department of Anesthesiology, Affiliated Hospital of Zunyi Medical University, 149#, Dalian Road, ZunyiGuizhou Province, 563000 China; 3 Department of Pathology, Affiliated Hospital of Zunyi Medical University, ZunyiGuizhou Province, 563000 China; 4 Department of Pediatrics, Affiliated Hospital of Zunyi Medical University, ZunyiGuizhou Province, 563000 China

**Keywords:** Colorectal cancer, Colorectal cancer surgery, Perioperative risk factors, Survival outcome, Sevoflurane, High-grade inflammation

## Abstract

**Background:**

Surgical resection remains the best option for long-term survival in colorectal cancer (CRC); however, surgery can lead to tumor cell release into the circulation. Previous studies have also shown that surgery can affect cancer cell growth. The role of perioperative factors influencing long-term survival in patients presenting for CRC surgery remains to be investigated.

**Methods:**

This retrospective single–center cohort study was conducted to collect the clinical data of patients who underwent elective laparoscopic resection for CRC from January 2014 to December 2015, namely clinical manifestations, pathological results, and perioperative characteristics. Survival was estimated using the Kaplan–Meier log-rank test. Univariable and multivariable Cox regression models were used to compare hazard ratios (HR) for death.

**Results:**

A total of 234 patients were eligible for analysis. In the multivariable Cox model, tumor-node-metastasis (TNM) stage (stage IV: HR 30.63, 95% confidence interval (CI): 3.85–243.65; P = 0.001), lymphovascular invasion (yes: HR 2.07, 95% CI 1.09–3.92; P = 0.027), inhalational anesthesia with isoflurane (HR 1.96, 95% CI 1.19–3.21; P = 0.008), and Klintrup–Makinen (KM) inflammatory cell infiltration grade (low-grade inflammation: HR 2.03, 95% CI 1.20–3.43; P = 0.008) were independent risk factors affecting 5-year overall survival after laparoscopic resection for CRC.

**Conclusions:**

TNM stage, lymphovascular invasion, isoflurane, and KM grade were independent risk factors affecting CRC prognosis. Sevoflurane and high-grade inflammation may be associated with improved survival in CRC patients undergoing resection.

## Background

Colorectal cancer (CRC) accounts for approximately 10% of all annually diagnosed cancers and cancer-related deaths worldwide. In 2018, over 1.8 million new CRC cases and 881,000 deaths were estimated. Overall, CRC ranks third in incidence but second in mortality, and currently presents a global public health problem [1, 2].

Surgical resection is the primary treatment for patients suffering from CRC [3]. Paradoxically, observations concerning the negative effects of surgery on cancer development have been documented 2000 years ago. It has been acknowledged that surgery itself may cause metabolic and neuroendocrine changes, thus inhibiting cell-mediated immunity, stimulating the implantation of circulating tumor cells, and leading to tumor recurrence or metastasis, and is associated with worse long-term outcomes [4–7]. Moreover, perioperative influencing factors, such as anesthetics, stress and inflammation, may be a decisive window, in which competitive factors promote or prevent the metastasis of residual cancer cells.

There is increasing evidence from animal and human cancer cell studies that anesthetics can affect the immune system in different ways [8–12], but few studies have compared sevoflurane-versus isoflurane-based anesthesia. Moreover, it is increasingly obvious that the prognosis of CRC does not entirely depend on the characteristics of the tumor. Tumor related inflammation has an unexpected and contradictory role in promoting tumor progression, and in effect helps early tumor formation to obtain the ability of markers [13]. Therefore, we conducted a retrospective study to explore prognostic factors affecting survival outcomes, and to assess whether the choice of anesthetics, sevoflurane versus isoflurane, and the effect of local or systemic inflammatory response are associated with long-term survival after elective laparoscopic colorectal resection.

## Methods

### Study design

This was a retrospective cohort study conducted at the Affiliated Hospital of Zunyi Medical University, Zunyi, Guizhou, China.

### Participants and data sources

**Fig. 1:**
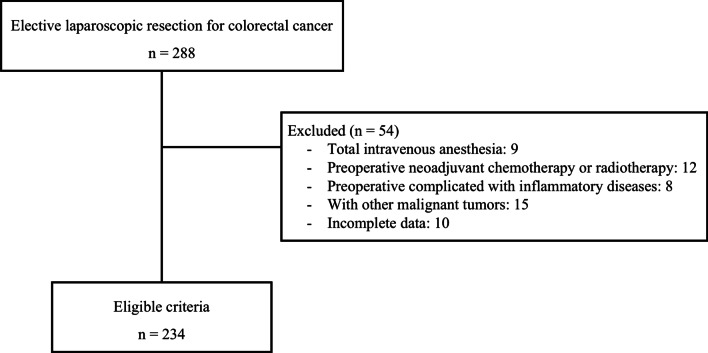
Flow diagram showing the selection of patients included in this retrospective analysis

The study was conducted in accordance with the Declaration of Helsinki and the study protocol was reviewed and approved by the Biomedical Research Ethics Committee of the Affiliated Hospital of Zunyi Medical University (Approval number: KLL-2020-266). Informed consent was waived by the the Biomedical Research Ethics Committee of the Affiliated Hospital of Zunyi Medical University. We constructed a database of retrospectively collected data from patients’ medical records from the Affiliated Hospital of Zunyi Medical University, including clinical characteristics, pathological reports, and survival during the follow-up period. From January 2014 to December 2015, 288 patients with an American Society of Anesthesiologists (ASA) score of II–III who underwent elective laparoscopic resection of CRC for tumor–node–metastasis (TNM) stage I–IV CRC according to the Union for International Cancer Control–American Joint Committee on Cancer 8^th^ edition under sevoflurane anesthesia or isoflurane anesthesia were considered for inclusion. The type of anesthesia was determined per the anesthesiologist’s preference. No desflurane or spinal anesthesia was used in these patients, and 54 patients were excluded from the analysis. The exclusion criteria were total intravenous anesthesia; preoperative neoadjuvant chemotherapy or radiotherapy; preoperatively complicated with inflammatory diseases; other malignant tumors; or incomplete data (Fig. 1).

### Variables

**Fig. 2:**
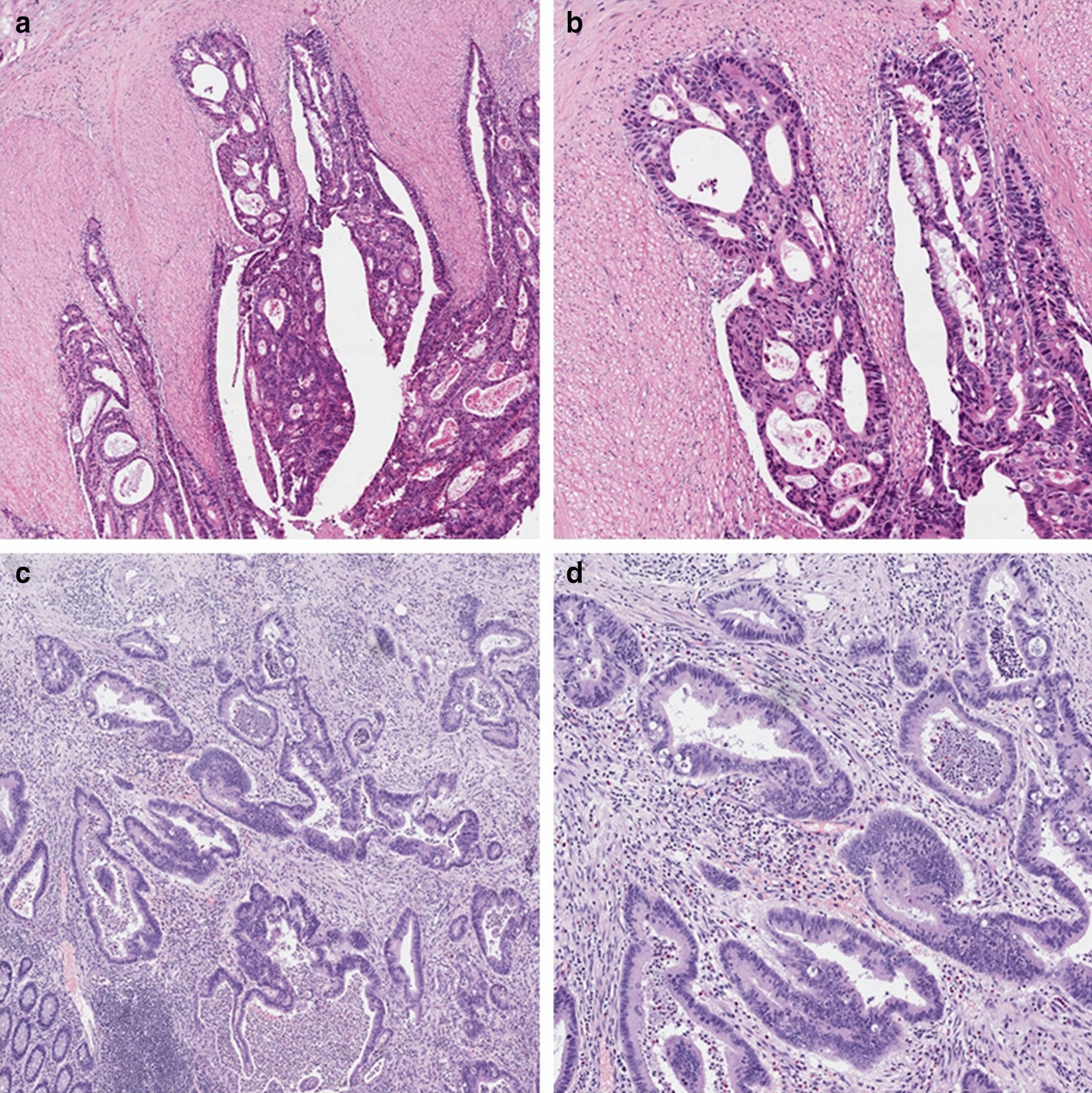
Infiltration of inflammatory cells at the tumor margin under optical microscopy. Images **a** and **b** indicate low-grade inflammatory cell infiltration (magnification: **a**, × 4; **b**, × 10), and images **c** and **d** indicate high-grade inflammatory cell infiltration (**c**, × 4; **d**, × 10)

We retrospectively collected the following patient data: sex; age at the time of surgery; weight; nationality; TNM stage; tumor location; tumor classification; histological type [ adenocarcinoma NOS (not otherwise specified), and special types of cancer (mucinous adenocarcinoma, signet ring cell carcinoma, medullary carcinoma, and neuroendocrine carcinoma)]; degree of differentiation; lymphovascular invasion; the ASA physical status (recorded by the anesthetist or the preassessment team preoperatively); inhalational anesthetic; anesthesia time; duration of surgery; postoperative analgesia; and postoperative hospitalization days. Preoperative systemic inflammatory response was evaluated by inflammation-based prognostic (IPS) scores [14] (Table 1). Inflammatory cell response at the cancer invasive border of the histopathological specimens was assessed by Klintrup–Makinen (KM) grade [15]. A score of 0 denoted no increase in inflammatory cells, and 1 denoted mild and patchy increase in inflammatory cells at the invasive margin, but no destruction of invading cancer cell islets by the inflammatory cells. A score of 2 denoted inflammatory cells forming a band-like infiltrate at the invasive margin with some destruction of cancer cell islets by inflammatory cells. A score of 3 denoted a very prominent inflammatory reaction forming a cup-like zone at the invasive margin, and destruction of cancer cell islets was frequent and invariably present. Furthermore, mild increase (scores 0–1) were combined as low-grade inflammation and moderate to strong increase (scores 2–3) as high-grade inflammation (Fig. 2). These variables were chosen as potential risk factors as they have either been shown, or are posited, to affect survival outcomes.

**Table 1:** Inflammation-based prognostic scores

Scoring system	Score
*Neutrophil*–*lymphocyte ratio (NLR)*	
Neutrophil count: lymphocyte count ≥ 5	1
Neutrophil count: lymphocyte count < 5	0
*Derived neutrophil*–*lymphocyte ratio (dNLR)*	
Neutrophil count: (leucocyte count–neutrophil count) ≥ 3	1
Neutrophil count: (leucocyte count–neutrophil count) < 3	0
*Lymphocyte*–*monocyte ratio (LMR)*	
Lymphocyte count: monocyte count < 2.35	1
Lymphocyte count: monocyte count ≥ 2.35	0
*Platelet*–*lymphocyte ratio (PLR)*	
Platelet count: lymphocyte count > 300	2
Platelet count: lymphocyte count 150–300	1
Platelet count: lymphocyte count < 150	0
*Combination of platelet count and NLR (COP*–*NLR)*	
NLR > 3 and platelet count > 300 × 10^9^/L	2
NLR > 3 or platelet count > 300 × 10^9^/L	1
Neither NLR > 3 nor platelet count > 300 × 10^9^/L	0
*Prognostic nutritional index (PNI)*	
Albumin (g/L) + 5 × total lymphocyte count (× 10^9^/L) < 45	1
Albumin (g/L) + 5 × total lymphocyte count (× 10^9^/L) ≥ 45	0

The primary endpoint was overall survival (OS). Survival time was defined as the interval between the date of surgery and the date of death or June 10, 2020, for those who were censored.

### Statistical analysis

Statistical analysis was performed using the Statistical Package for Social Sciences (SPSS™), Windows version 23.0 (IBM Corp., Armonk, NY, USA). All data are presented as n (%).

Five-year OS was depicted visually as a Kaplan–Meier survival curve, and the log-rank test was used to analyze univariable distributions for OS. We used a Cox proportional hazards model for univariable and multivariable analyses to calculate hazard ratios (HR) and corresponding 95% confidence intervals (95% CI). Statistical significance was accepted for P values < 0.05.

## Results

From a total of 288 patients operated for CRC from 2014 to 2015, 234 were included in this study. All patients were followed-up regularly until June 10, 2020 or until death. The median follow-up period was 56.5 months (range 30 days to 76 months); 85 (36.3%) patients died in this cohort. The median age at the time of surgery was 60.5 years (range 29–87 years), and the male:female ratio was 131 (56.0%):103 (44.0%). The most common TNM stage was III (58.5%). Of the 234 patients, 104 (44.4%) received sevoflurane anesthesia, and 130 (55.6%) received isoflurane. The frequencies of low- and high-grade inflammation were 89 (38.0%) and 145 (62.0%), respectively. Patient and perioperative characteristics are shown in Table 2.

**Table 2:** Patients’ clinical and perioperative characteristics

Variables	N (%)
*Sex*	
Male	131 (56.0)
Female	103 (44.0)
*Age (years)*	
< 60	109 (46.6)
≥ 60	125 (53.4)
*Weigh (kg)*	
< 55	113 (48.3)
≥ 55	121 (51.7)
*Nationality*	
The Han	217 (92.7)
Minority nationality	17 (7.3)
*Tumor location*	
Rectum	182 (77.8)
Colon	52 (22.2)
*TNM stage*	
I	15 (6.4)
II	59 (25.2)
III	137 (58.5)
IV	23 (9.8)
*Tumor classification*	
The elevated	88 (37.6)
The ulcerative	110 (47.0)
The infiltrative	36 (15.4)
*Histological type*	
Adenocarcinoma (NOS)	196 (83.8)
Special types of cancer	38 (16.2)
*Degree of differentiation*	
High	63 (26.9)
Medium	140 (59.8)
Low	31 (13.2)
*Lymphovascular invasion*	
Yes	25 (10.7)
No	209 (89.3)
*ASA*	
II	194 (82.9)
III	40 (17.1)
*Inhalation anesthetics*	
Sevoflurane	104 (44.4)
Isoflurane	130 (55.6)
*Anesthesia time (h)*	
< 4.5	110 (47.0)
≥ 4.5	124 (53.0)
*Duration of surgery (h)*	
< 3.5	122 (52.1)
≥ 3.5	112 (47.9)
*Postoperative analgesia*	
Yes	227 (97.0)
No	7 (3.0)
*Postoperative hospitalization days*	
< 13	117 (50.0)
≥ 13	117 (50.0)
*IPS score*	
< 1	78 (33.3)
≥ 1	156 (66.7)
*KM grade*	
Low–grade inflammation	89 (38.0)
High–grade inflammation	145 (62.0)

Data shown as n (%)

*TNM* tumor-node-metastasis, *Adenocarcinoma (NOS)* adenocarcinoma, not otherwise specified, *ASA* American Society of Anesthesiologists, *IPS score* inflammation-based prognostic scores, *KM grade* Klintrup–Makinen (KM) inflammatory cell infiltration grade

Associations between each variable and OS are presented in Table 3, for which univariable analysis and multivariable Cox regression were performed. Significant variables (TNM stage, histological type, lymphovascular invasion, inhalational anesthetic, and KM grade) in the univariable analysis were included in the multivariable Cox proportional hazards model. The multivariable analysis indicated that TNM stage (stage IV: HR 30.63, 95% CI 3.85–243.65; P = 0.001), lymphovascular invasion (yes: HR 2.07, 95% CI 1.09–3.92; P = 0.027), inhalational anesthesia with isoflurane (HR 1.96, 95% CI 1.19–3.21; P = 0.008), and KM grade (low-grade inflammation: HR 2.03, 95% CI 1.20–3.43; P = 0.008) were independent prognostic factors for 5-year OS.

**Table 3:** Cox proportional hazards regression analysis of mortality: univariable and multivariable models for the entire cohort

Variables	Univariable		Multivariable	
	HR (95% CI)	*P* value	HR (95% CI)	*P* value
*Sex*				
Male	1		1	
Female	0.86 (0.56–1.33)	0.506	1.13 (0.69–1.85)	0.621
*Age (years)*				
< 60	1		1	
≥ 60	0.76 (0.50–1.16)	0.201	0.62 (0.38–1.01)	0.057
*Weigh (kg)*				
< 55	1		1	
≥ 55	1.01 (0.66–1.54)	0.982	0.74 (0.46–1.18)	0.210
*Nationality*				
The Han	1		1	
Minority nationality	1.26 (0.58–2.73)	0.560	0.97 (0.41–2.32)	0.950
*Tumor location*				
Rectum	1		1	0.103
Colon	0.61 (0.34–1.10)	0.100	0.59 (0.31–1.11)	
*TNM stage*				
I	1		1	
II	3.87 (0.51–29.46)	0.191	3.39 (0.43–26.76)	0.246
III	6.41 (0.89–46.48)	0.066	4.54 (0.60–34.55)	0.144
IV	42.55 (5.67–319.14)	< **0.001**	30.63 (3.85–243.65)	**0.001**
*Tumor classification*				
The elevated	1		1	
The ulcerative	1.32 (0.81–2.15)	0.273	1.46 (0.85–2.52)	0.172
The infiltrative	1.82 (1.00–3.32)	0.052	0.40 (0.06–2.91)	0.368
*Histological type*				
Adenocarcinoma (NOS)	1		1	
Special types of cancer	1.69 (1.01–2.81)	**0.042**	4.28 (0.55–33.31)	0.165
*Degree of differentiation*				
High	1		1	
Medium	1.03 (0.62–1.71)	0.923	1.17 (0.66–2.08)	0.592
Low	1.58 (0.81–3.06)	0.180	0.94 (0.40–2.20)	0.886
*Lymphovascular invasion*				
No	1		1	
Yes	1.95 (1.10–3.46)	**0.020**	2.07 (1.09–3.92)	**0.027**
*ASA*				
II	1		1	
III	1.15 (0.67–1.98)	0.614	0.86 (0.47–1.59)	0.632
*Inhalation anesthetics*				
Sevoflurane	1		1	
Isoflurane	1.68 (1.07–2.65)	**0.022**	1.96 (1.19–3.21)	**0.008**
*Anesthesia time (h)*				
< 4.5	1		1	
≥ 4.5	0.99 (0.65–1.52)	0.976	1.02 (0.47–2.22)	0.964
*Duration of surgery (h)*				
< 3.5	1		1	
≥ 3.5	0.97 (0.64–1.49)	0.902	0.85 (0.38–1.88)	0.686
*Postoperative analgesia*				
No	1		1	
Yes	0.52 (0.19–1.41)	0.196	0.44 (0.14–1.34)	0.149
*Postoperative hospitalization days*				
< 13	1		1	
≥ 13	1.26 (0.82–1.93)	0.293	1.20 (0.73–1.97)	0.471
*IPS score*				
< 1	1		1	
≥ 1	1.26 (0.79–2.00)	0.326	1.19 (0.72–1.98)	0.496
*KM grade*				
High–grade inflammation	1		1	
Low–grade inflammation	2.05 (1.34–3.15)	**0.001**	2.03 (1.20–3.43)	**0.008**

Bold values indicate statistically significant (P < 0.05)

*HR* hazard ratios, *CI* confidence interval, *TNM* tumor–node–metastasis, *Adenocarcinoma (NOS)* adenocarcinoma, not otherwise specified, *ASA* American Society of Anesthesiologists, *IPS score* inflammation-based prognostic scores, *KM grade* Klintrup–Makinen (KM) inflammatory cell infiltration grade

**Fig. 3:**
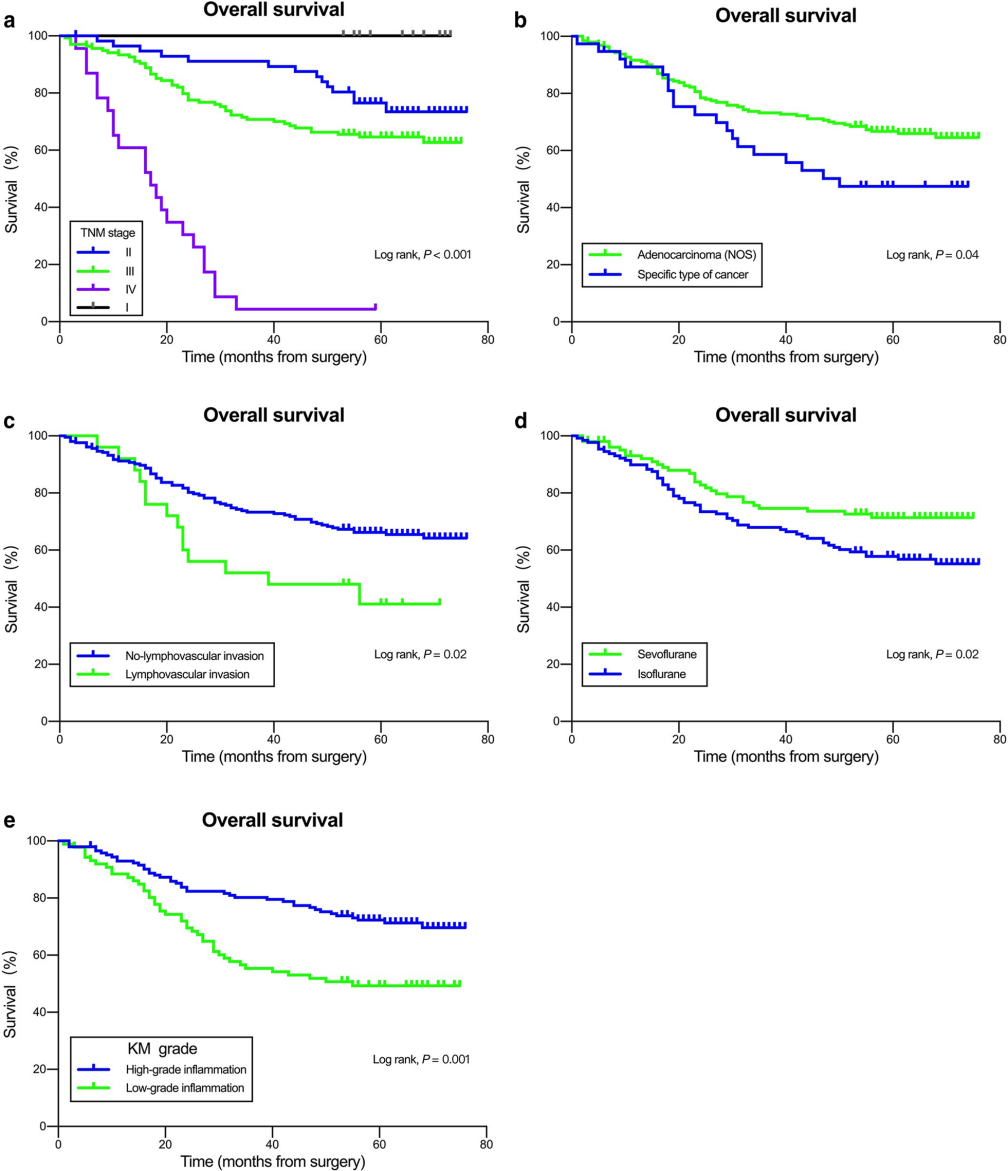
Kaplan–Meier survival curves for patients with colorectal cancer. **a** Tumor–node–metastasis (TNM) stage; **b** histological type; **c** lymphovascular invasion; **d** inhalational anesthetic type; **e** Klintrup–Makinen inflammatory cell infiltration grade (KM grade)

**Fig. 4:**
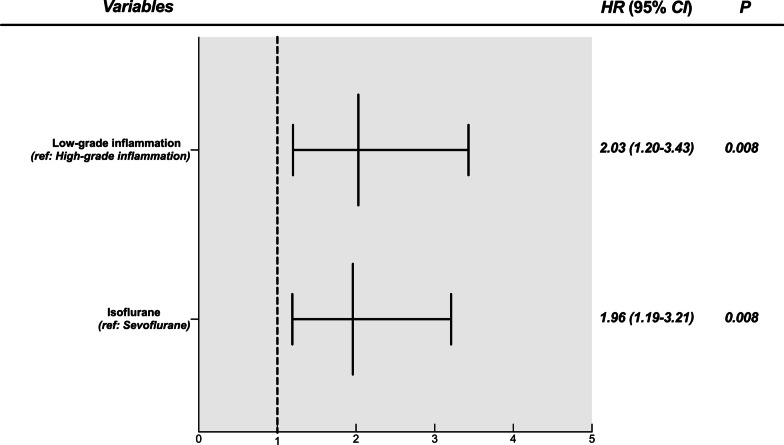
Forest plot of the survival analysis showing multivariable hazard ratios. This analysis demonstrated significantly worse outcomes in patients with low-grade inflammation, or those receiving isoflurane inhalational anesthesia

The Kaplan–Meier OS curves according to TNM stage, histological type, lymphovascular invasion, inhalational anesthetics, and KM grade are shown in Fig. 3. Forest plots indicated that isoflurane inhalational anesthesia (P = 0.008) and low-grade inflammation (P = 0.008) were independently associated with a high risk of death after operation (Fig. 4).

## Discussion

This retrospective analysis of 234 patients who underwent elective laparoscopic CRC resection over a 5-year period evaluated long-term survival in patients receiving sevoflurane- versus isoflurane-based anesthesia. After analysis using a Cox proportional hazards model, our results suggested that a beneficial effect of sevoflurane inhalational anesthesia and high-grade inflammation for CRC surgery patients was associated with better survival compared with isoflurane anesthesia and low-grade inflammation, respectively. There was a significant association between sevoflurane and high-grade inflammation, and improved survival. IPS score was not associated with a better outcome. We identified associations between TNM stage and lymphovascular invasion, and overall survival after CRC surgery. These findings were consistent with those of prior observational studies that evaluated other types of cancers, such as breast, esophageal squamous cell carcinoma, and gastric cancer [16–18], and showed that these variables decreased the survival of patients after CRC resection. In our study, we focused on overall survival in patients receiving inhalational anesthetics, and the relationship between survival and pathological KM grade after laparoscopic CRC resection. However, previous studies mainly indicated that propofol-based intravenous anesthesia was associated with improved survival in cancer patients undergoing resection [7, 19–21]. To our knowledge, few studies have systematically compared the effects of isoflurane and sevoflurane on the development of CRC after laparoscopic CRC resection. In our study, we found that sevoflurane was superior to isoflurane in lowering mortality after laparoscopic CRC resection.

The perioperative period is of great interest regarding the risk for recurrence after surgery for CRC. There are many factors to promote the postoperative metastasis and recurrence of primary tumors. Tumor cell proliferation and postoperative immunosuppression are considered to be related to tumor metastasis and recurrence. The immune system, especially the cellular immune response, can prevent the proliferation of cancer cells, and plays a central role in the removal of cancer cells after surgery, which is inhibited by surgery. In our study, we also found that high-grade inflammation was associated with superior survival after colorectal surgery for CRC, which was consistent with most published studies suggesting that tumor inflammation or immune cell infiltration in patients with CRC could be a good prognostic marker for CRC [22, 23]. Some studies also show that tumor immune invasion is associated with good prognosis, including survival, recurrence and metastasis rate [24, 25]. However, there may be a variety of immune cell types in a tumor, including innate and adaptive immune cells, and different immune cell subsets in different tumor types. Further prospective studies will help to understand the effect of immune cell subsets on recurrence and metastasis of CRC, to inform improved management and pre-operative optimisation of patients undergoing surgery [26]. At the same time, continuous education related to decision-making and practical handson courses is also necessary [27].

Several limitations should be considered when interpreting the results in our study. First, this was retrospective study, and patients were not randomly allocated. Certain perioperative confounding factors, such as the specific drugs administered, detailed surgical techniques, surgical complications, and intraoperative blood transfusion, also introduced bias. Second, propofol anesthesia has been linked to better survival in cancer populations. In our hospital, we routinely use propofol during CRC resection, and propofol was administered to all patients enrolled in the study; therefore, there was no difference between groups regarding propofol, in the current study. Third, we did not consider the quality and the type of analgesia (e.g., different opioids or analgesic protocols) because 97% of the patients in our study received patient-controlled intravenous analgesia, and the surgeons administered additional analgesics when patients complained of pain. A future prospective study would be useful to validate our conclusions. Definitive evidence of a causal link would have to come from a prospective trial.

## Conclusions

In conclusion, we found significantly lower mortality in patients receiving sevoflurane anesthesia for CRC, compared with those receiving isoflurane anesthesia. Additionally, high-grade inflammation was associated with better survival compared with low-grade inflammation. Prospective, randomized studies are needed to determine the exact role of sevoflurane and inflammation in preventing cancer-related morbidity and mortality after CRC surgery.

## Data Availability

The datasets used and/or analysed during the current study are available from the corresponding author (ZQZ) on reasonable request.

## Abbreviations

CRC: Colorectal cancer
TNM: Tumor-node-metastasis
HR: Hazard ratios
CI: Confidence interval
KM grade: Klintrup–Makinen inflammatory cell infiltration grade
ASA: An American Society of Anesthesiologists
Adenocarcinoma (NOS): Adenocarcinoma (not otherwise specified)
IPS: Inflammation-based prognostic
OS: Overall survival
